# Supplementary material for: Electrotaxis evokes directional separation of co-cultured keratinocytes and fibroblasts
Source: Sci Rep. 2023 Jul 15;13:11444. doi: 10.1038/s41598-023-38664-y (PMC10349865; doi:10.1038/s41598-023-38664-y)
Supplement: Supplementary file 1 — Supplementary Tables. [file 41598_2023_38664_MOESM1_ESM.pdf]

Table ST1: List of publications related to keratinocyte electrotaxis. PubMed search: "Keratinocytes AND (electrotaxis OR galvanotaxis OR electric field)

	Title	Year	Stimulation paradigm	EF in mV/mm	Cell type	Research type	Focus	Modified parameters	Reference
1	Parting the cellular sea: electrotaxis evoked directional separation of co-cultured keratinocytes and fibroblasts	2023	**DC**	**50,100,200,400**	Human Dermal Fibroblasts	Cell migration<br>Co-culture with keratinocytes	**Engineering/ Biology**	EF strength<br>EF direction<br>Cell Media + co-culture with keratinocytes	This Manuscript
2	Bioelectronic microfluidic wound healing: a platform for investigating direct current stimulation of injured cell collectives.	2023	**DC**	**200**	Primary Human Keratinocytes - monolayer	Wound closure	**Engineering**	EF direction<br>Media composition (diabetes)	Lab Chip. 2023 Mar 14;23(6):1531-1546. doi: 10.1039/d2lc01045c.
3	Engineering diabetic human skin equivalent for in vitro and in vivo applications.	2022	**DC**	**20 , 40**	Diabetic Keratinocytes & Fibroblasts - engineered tissue	Cell activity & proliferation	**Engineering**	EF strength	Front Bioeng Biotechnol. 2022 Sep 30;10:989888. doi:10.3389/fbioe.2022.989888. eCollection 2022.
4	Come together: On-chip bioelectric wound closure	2021	**DC**	**200**	Primary Human Keratinocytes - monolayer	Wound closure	**Engineering**		Biosens Bioelectron. 2021 Nov 15;192:113479. doi: 10.1016/j.bios.2021.113479. Epub 2021 Jul 8.
5	Overriding native cell coordination enhances external programming of collective cell migration	2021	**DC**	**200**	Primary Mouse Keratinocytes - monolayer	Monolayer migration	**Biology**	Ca2+ concentration<br>Anti-E Cadherin antibody (DECMA)<br>BAPTA	Proc Natl Acad Sci U S A. 2021 Jul 20;118(29):e2101352118. doi: 10.1073/pnas.2101352118
6	SCHEEPDOG: Programming Electric Cues to Dynamically Herd Large-Scale Cell Migration	2020	**DC**	**200**	Primary Human Keratinocytes - monolayer	Cell control in EF	**Engineering**	EF direction in X and Y	Cell Syst. 2020 Jun 24;10(6):506-514.e3. doi: 10.1016/j.cels.2020.05.009. Epub 2020 Jun
7	Electric field down-regulates CD9 to promote keratinocytes migration through AMPK pathway.	2020	**DC**	**200**	HaCaT Keratinocytes - single cells	EF influence on genetics	**Biology**	CD9 overexpression	Int J Med Sci. 2020 Mar 15;17(7):865-873. doi: 10.7150/ijms.42840. eCollection 2020
8	Keratinocyte electrotaxis induced by physiological pulsed direct current electric fields.	2019	**Pulsed DC**	**150**	Primary Mouse Keratinocytes - single cells	EF influence on electrotaxis	**Biology**		Bioelectrochemistry. 2019 Jun;127:113-124. doi: 10.1016/j.bioelechem.2019.02.001. Epub 2019 Feb 14.
9	Electric field-induced migration and intercellular stress alignment in a collective epithelial monolayer.	2018	**DC**	**50**	HaCaT Keratinocytes - monolayer	Monolayer migration	**Biology**	EF directedness	Mol Biol Cell. 2018 Sep 15;29(19):2292-2302. doi: 10.1091/mbc.E18-01-0077. Epub 2018 Jul 25.
10	Electric field-induced suppression of PTEN drives epithelial-to-mesenchymal transition via mTORC1 activation.	2017	**DC**	**100**	Primary Human Keratinocytes - monolayer	Wound closure	**Biology**	Transfection with PTEN	J Dermatol Sci. 2017 Feb;85(2):96-105. doi:10.1016/j.jdermsci.2016.11.007. Epub 2016 Nov 18.
11	Kindlin-1 Regulates Keratinocyte Electrotaxis.	2016	**DC**	**50 , 100 , 200**	Immortalized Human Keratinocytes - FERMT1mutated	Influence of Kindlin 1 on EF sensing	**Biology**	Presence of Kindlin 1	J Invest Dermatol. 2016 Nov;136(11):2229-2239. doi:10.1016/j.jid.2016.05.129. Epub 2016 Jul 15.
12	Spontaneous and electric field-controlled front-rear polarization of human keratinocytes.	2015	**DC**	**100**	Immortalized Human Keratinocytes - single cells	Cell polarization	**Biology**	pH<br>Drugs - Suramin, PTX, PPADS, Gallein	Mol Biol Cell. 2015 Dec 1;26(24):4373-86. doi: 10.1091/mbc.E14-12-1580. Epub 2015 Sep 30.
13	ATP Release and P2 Y Receptor Signaling are Essential for Keratinocyte Galvanotaxis.	2016	**DC**	**100**	Primary Human Keratinocytes - single cells	Influence of ATP on EF sensing	**Biology**	Suramin<br>P2Y receptor	J Cell Physiol. 2016 Jan;231(1):181-91. doi: 10.1002/jcp.25070.
14	The Galvanotactic Migration of Keratinocytes is Enhanced by Hypoxic Preconditioning.	2015	**DC**	**25 , 50 , 100 , 200**	Primary Mouse Keratinocytes - single cells + monolayers	Influence of Oxygen + EF	**Biology**	oxygen saturation	Sci Rep. 2015 May 19;5:10289. doi: 10.1038/srep10289.
15	Improvement of human keratinocyte migration by a redox active bioelectric dressing.	2014	**DC**	**≤ 270**	HaCaT Keratinocytes - monolayer	Influence of EF on electrotyxis + H2O2	**Engineering/ Biology**	Number of Ag/Zn dots	PLoS One. 2014 Mar 3;9(3):e89239. doi: 10.1371/journal.pone.0089239. eCollection 2014.
16	The epithelial sodium channel mediates the directionality of galvanotaxis in human keratinocytes.	2013	**DC**	**100**	Human Keratinocytes<br>Mouse Keratinocytes aENaC	Influence of ENaC on EF sensing	**Biology**	Supression or expression of ENaC	J Cell Sci. 2013 May 1;126(Pt 9):1942-51. doi: 10.1242/jcs.113225. Epub 2013 Feb 27.
17	Keratinocyte galvanotaxis in combined DC and AC electric fields supports an electromechanical transduction sensing mechanism.	2013	**DC + AC**	**100 (DC)<br>40 (AC) 1.6,160 Hz**	Primary Human Keratinocytes - single cells	Electromechanical sensing of EF	**Biology**	AC frequency + AC-DC superposition	Bioelectromagnetics. 2013 Feb;34(2):85-94. doi: 10.1002/bem.21748. Epub 2012 Aug 21.
18	Human keratinocytes respond to direct current stimulation by increasing intracellular calcium: preferential response of poorly differentiated cells.	2012	**DC**	**200<br>100,200,400,900**	Primary Human Keratinocytes - monolayer	Influence of EF on intracellular Ca2+	**Biology**	Ca2+ concentration<br>Voltage Gated Ca2+ Channels blockers	J Cell Physiol. 2012 Jun;227(6):2660-7. doi: 10.1002/jcp.23008.
19	Electrical signals control wound healing through phosphatidylinositol-3-OH kinase-γ and PTEN	2006	**DC**	**100, 150, 200**	Primary Mouse Keratinocytes - monolayer<br>Ex vivo cornea cultures	Influence of PI3K and PTEN on EF sensign	**Biology**		Nature. 2006 Jul 27;442(7101):457-60. doi: 10.1038/nature04925.
20	beta4 integrin and epidermal growth factor coordinately regulate electric field-mediated directional migration via Rac1.	2006	**DC**	**100**	Primary Human Keratinocytes - single cells<br>Keratinocytes lacking beta4 integrin	Influence of beta4 integrin in EF sensing	**Biology**	Retroviral transduction to express or not beta 4	Mol Biol Cell. 2006 Nov;17(11):4925-35. doi: 10.1091/mbc.e06-05-0433. Epub 2006 Aug 16.
21	The Ras/Raf-1/MEK1/ERK signaling pathway coupled to integrin expression mediates cholinergic regulation of keratinocyte directional migration.	2005	**DC**	**100**	Human Keratinocytes - single cells	Influence of various signaling pathways on celular orientation in an EF	**Biology**	Chemotaxis<br>Galvanotropism<br>Activated pathway	J Biol Chem. 2005 Nov 25;280(47):39220-8. doi: 10.1074/jbc.M504407200. Epub 2005 Sep 8.

**22**	Cyclic AMP mediates keratinocyte directional migration in an electric field.	2005	**DC**	**100**	Human Keratinocytes - single cells	Importance of cyclic AMP on electrotaxis	**Biology**	AMP agonists / antagonists<br>beta2-AR, sp-cAMP, pertussis toxin, forskolin	J Cell Sci. 2005 May 1;118(Pt 9):2023-34. doi: 10.1242/jcs.02330. Epub 2005 Apr 19.
**23**	Calcium channel blockers inhibit galvanotaxis in human keratinocytes.	2002	**DC**	**100**	Human Keratinocytes - single cells	Influence of Ca2+ in Electrotaxis	**Biology**	Ca2+ channel blockers<br>amiloride, verapamil, gandolinium chloride, strontium chloride, nickel chloride	J Cell Physiol. 2002 Oct;193(1):1-9. doi: 10.1002/jcp.10144.
**24**	Cyclic AMP-dependent protein kinase A plays a role in the directed migration of human keratinocytes in a DC electric field.	2001	**DC**	**100**	Human Keratinocytes - single cells	Influence of cyclic AMP on electrotaxis	**Biology**	Concentration of kinase inhibitor<br>KT5720, W-7, Sphignosine, GF109203X, ML-7, H-7	Cell Motil Cytoskeleton. 2001 Dec;50(4):207-17. doi: 10.1002/cm.10009.
**25**	Epidermal growth factor receptor relocalization and kinase activity are necessary for directional migration of keratinocytes in DC electric fields	1999	**DC**	**100**	Human Keratinocytes - single cells	Effects of protein tyrosine kinase inhibitors on electrotaxis	**Biology**	Type and concentration of kinase inhibitor<br>PD158780, Genistein, Lavendustin A, Tyrphostin B4	J Cell Sci. 1999 Jun;112 (Pt 12):1967-78. doi: 10.1242/jcs.112.12.1967.
**26**	Involucrin-positive keratinocytes demonstrate decreased migration speed but sustained directional migration in a DC electric field.	1999	**DC**	**100**	Human Keratinocytes - single cells	Influence of involucrin expresssion on EF sensing	**Biology**	Involucrin positive vs negative	J Invest Dermatol. 1999 Nov;113(5):851-855. doi: 10.1046/j.1523-1747.1999.00763.x.
**27**	Migration of human keratinocytes in electric fields requires growth factors and extracellular calcium.	1998	**DC**	**100**	Human Keratinocytes - single cells	Influence of extracellular Ca2+ and growth factors on electrotaxis	**Biology**	Concentration of: HKGS, EGF, Insulin, BPE	J Invest Dermatol. 1998 Nov;111(5):751-6. doi: 10.1046/j.1523-1747.1998.00366.x.
**28**	Imposition of a physiologic DC electric field alters the migratory response of human keratinocytes on extracellular matrix molecules.	1996	**DC**	**100, 400**	Human Keratinocytes - single cells	Influence of extracellular matrix on electrotaxis	**Biology**	Type of substrate coating<br>collagen I, collagen IV, fibronectin, laminin, plastic	J Invest Dermatol. 1996 Apr;106(4):642-6. doi: 10.1111/1523-1747.ep12345456.
**29**	Human keratinocytes migrate to the negative pole in direct current electric fields comparable to those measured in mammalian wounds.	1996	**DC**	**10,20,50,100,200,400**	Human Keratinocytes - single cells	Influence of EF magnitude on cell migration	**Biology**	EF magnitude	J Cell Sci. 1996 Jan;109 (Pt 1):199-207. doi: 10.1242/jcs.109.1.199.

Table ST2: List of publications related to fibroblast electrotaxis. PubMed search: “Dermal Fibroblasts AND (electrotaxis OR galvanotaxis OR electric field)

	Title	Year	Stimulation paradigm	EF in mV/mm	Cell type	Research type	Focus	Modified parameters	Reference
**1**	Parting the cellular sea: electrotaxis evoked directional separation of co-cultured keratinocytes and fibroblasts	2023	**DC**	**50,100,200,400**	Human Dermal Fibroblasts	Cell migration<br>Co-culture with keratinocytes	**Engineering/ Biology**	EF strength<br>EF direction<br>Cell Media + co-culture with keratinocytes	This Manuscript
**2**	Pulse Capacitive Coupling Electric Field Regulates Cell Migration, Proliferation, Polarization, and Vascularization to Accelerate Wound Healing.	2023	**Pulsed capacitive coupling EF**	**54 @ 60Hz**	Human Dermal Firbroblasts<br>Primari Keratinocytes	Cell migration	**Engineering**		Adv Wound Care (New Rochelle). 2023 Jan 18. doi: 10.1089/wound.2021.0194. Online ahead of print.
**3**	Electrical Shunting Prevents the Decline of Galvanotaxis After Monophasic Pulsed Microcurrent Stimulation in Human Dermal Fibroblasts.	2022	**DC with shunt**	**N/A**	Primary Human Dermal Fibroblasts	Cell migration	**Engineering**	Stimulation current<br>*100, 200, 400 µA	Eplasty, 22, e27.
**4**	A pulsed current electric field alters protein expression creating a wound healing phenotype in human skin cells.	2020	**DC<br>Pulsed DC**	**100<br>50 mA 128 Hz**	Human Dermal Firbroblasts<br>Normal Keratinocytes<br>Microvascular Endothelial cells	Cell Migration<br>Cell Proliferation<br>Gene Expression	**Engineering**	DC vs Pulsed DC	Regen Med. 2020 May;15(5):1611-1623. doi: 10.2217/rme-2019-0087. Epub 2020 Jul 7.
**5**	Monophasic Pulsed 200-µA Current Promotes Galvanotaxis With Polarization of Actin Filament and Integrin α2β1 in Human Dermal Fibroblasts.	2016	**Monophasic pulsed micro current**	**N/A**	Human Dermal Fibroblasts	Cell Migration<br>Cell Proliferation	**Engineering/ Biology**	Stimulation current<br>*100, 200, 300 µA	Eplasty. 2016 Jan 19;16:e6. eCollection 2016.
**6**	Golgi polarization plays a role in the directional migration of neonatal dermal fibroblasts induced by the direct current electric fields.	2015	**DC**	**100**	Human Dermal Fibroblasts	Cell migration<br>Influence of Golgi polarization on EF sensing	**Biology**	Influence of Brefeldin A (BFA)	Biochem Biophys Res Commun. 2015 May 1;460(2):255-60. doi: 10.1016/j.bbrc.2015.03.021. Epub 2015 Mar 13
**7**	Correlation between cell migration and reactive oxygen species under electric field stimulation	2015	**DC**	**100, 200, 400**	NIH 3T3 fibroblasts	Cell migration<br>influence of ROS	**Engineering/ Biology**	EF strength<br>addition of beta-lapachone	Biomicrofluidics. 2015 Oct 6;9(5):054120. doi: 10.1063/1.4932662.
**8**	Calcium Ion Flow Permeates Cells through SOCs to Promote Cathode-Directed Galvanotaxis	2015	**DC<br>Pulsed DC**	**500**	Mouse Dermal Fibroblasts	Cell Migration<br>Ca2+ flow	**Engineering/ Biology**	Ion flow at different EF strengths<br>Pulse frequency -0.1, 10, 1000 Hz<br>Duty cycle - 50%, 75%	PLoS One. 2015 Oct 8;10(10):e0139865. doi: 10.1371/journal.pone.0139865.
**9**	Control of neonatal human dermal fibroblast migration on poly(lactic-co-glycolic acid)-coated surfaces by electrotaxis.	2015	**DC**	**50, 100, 150, 200**	Human Dermal Fibroblasts	Cell Migration	**Biology**	Coating of surface with PLGA<br>EF intensity, EF direction	J. Tissue Eng Regen Med. 2017 Mar;11(3):862-868. doi: 10.1002/term.1986. Epub 2015 Jan 28.
**10**	Collaborative effects of electric field and fluid shear stress on fibroblast migration.	2013	**DC**	**50, 100, 200**	Human Dermal Fibroblasts	Cell Migration	**Engineering**	EF strength<br>Fluid shear velocity	Lab Chip. 2013 Apr 21;13(8):1602-11. doi: 10.1039/c3lc41240g.
**11**	Regeneration and control of human fibroblast cell density by intermittently delivered pulsed electric fields.	2013	**Pulsed DC**	**150<br>1 Hz**	Human Dermal Fibroblasts	Cell Proliferation	**Engineering/ Biology**	Number of stimulation pulses	Biotechnol Bioeng. 2013 Jun;110(6):1759-68. doi: 10.1002/bit.24831. Epub 2013 Mar 4.
**12**	Optimum microcurrent stimulation intensity for galvanotaxis in human fibroblasts.	2012	**Monophasic pulsed micro current**	**N/A**	Human Dermal Fibroblasts	Cell Migration	**Engineering/ Biology**	Stimulation Current<br>*50, 100 µA @ 0.3 Hz, 250 ms	J Wound Care. 2012 Jan;21(1):5-6, 8,10; discussion 10-1. doi: 10.12968/jowc.2012.21.Sup9.S5.
**13**	Effects of physiological electric fields on migration of human dermal fibroblasts.	2010	**DC**	**50, 100, 400**	Human Dermal Fibroblasts<br>Primary Dermal Fibroblasts	Cell Migration	**Biology**	EF strength<br>PI3K inhibition - LY294002	J Invest Dermatol. 2010 Sep;130(9):2320-7. doi: 10.1038/jid.2010.96. Epub 2010 Apr 22.
**14**	Human dermal fibroblasts do not exhibit directional migration on collagen I in direct-current electric fields of physiological strength.	2003	**DC**	**100**	Human Dermal Fibroblasts	Cell Migration	**Biology**	Cell media<br>Keratinocyte Media, Serum containing media (SM), High Mg2+ media (HM)	Exp Dermatol. 2003 Aug;12(4):396-402. doi: 10.1034/j.1600-0625.2002.120406.x.
**15**	Electric fields and proliferation in a chronic wound model.	1996	**AC**	**0.02 - 1**	Human Dermal Fibroblasts in collgaen matrix (3D)	Cell Proliferation<br>DNA changes	**Biology**	EF strength<br>frequency - 10, 100 Hz	Bioelectromagnetics. 1996;17(6):450-7. doi: 10.1002/(SICI)1521-186X(1996)17:6<450::AID-BEM4>3.0.CO;2-2
